# Physiological Mechanisms of Citrus Fruit Cracking: Study on Cell Wall Components, Osmoregulatory Substances, and Antioxidant Enzyme Activities

**DOI:** 10.3390/plants13020257

**Published:** 2024-01-16

**Authors:** Shengjia Huang, Xinxia Yang, Tie Wang, Hang Li, Lijun Deng, Xiaoyi Bi, Juan Hu, Yan Gong, Yunjie Li, Zeyu Qin, Yuan Yao, Guochao Sun, Ling Liao, Mingfei Zhang, Siya He, Lingping Jiang, Zhihui Wang

**Affiliations:** 1 College of Horticulture, Sichuan Agricultural University, Chengdu 611130, China; 2 Institute of Economic Forest Research, Sichuan Academy of Forestry, Chengdu 610081, China; 3 Institute of Pomology and Olericulture, Sichuan Agricultural University, Chengdu 611130, China; 4 Agricultural and Rural Bureau, Shimian, Ya’an 625400, China

**Keywords:** ‘Mingrijian’, citrus, fruit cracking, cell wall component, osmoregulation, antioxidant enzyme

## Abstract

Fruit cracking affects both the yield and economic efficiency of citrus; however, the underlying mechanism remains unclear. Therefore, this study focused on resistant and susceptible cultivars to identify the mechanisms underlying fruit cracking. The results showed that in ‘Mingrijian’, pectin morphological transformation and hemicellulose and lignin degradation in the pericarp were important contributing factors. During the critical fruit-cracking period (115–150 days after flowering), the water-soluble pectin, protopectin, and lignin contents in the pericarp of ‘Daya’ presented inverse changes relative to those in ‘Mingrijian’, thus enhancing the mechanical properties and resistance of pericarp. From 115 to 150 days after flowering, the soluble sugar content in the pulp of ‘Mingrijian’ increased rapidly by 97.35%, aiding in pulp water absorption and expansion. Moreover, the soluble protein content in the pericarp of ‘Mingrijian’ exhibited a declining trend and was lower than that of ‘Daya’, thus affecting the overall metabolism. The superoxide dismutase (SOD) activity in the pericarp of ‘Mingrijian’ gradually decreased from 115 to 180 days after flowering, while the peroxidase (POD) activity remained at a low level, resulting in weaker antioxidant capacity and lower environmental resistance. This study provides valuable insights into the mechanisms of citrus fruit cracking, laying the foundation for preventive and control strategies.

## 1. Introduction

*Citrus* belongs to the subfamily Aurantioideae and family Rutaceae and represents the world’s most widely grown and most productive fruit genus [1,2]. Fruit cracking is a physiological disorder in citrus fruit development that occurs in various citrus varieties, such as mandarins (*Citrus reticulata*), navel oranges (*Citrus sinensis*), and mandarin hybrids (*Citrus reticulata* × *Citrus sinensis* or *Citrus reticulata* × *Citrus paradise*), with some citrus varieties having a fruit cracking rate of up to 50% or more, resulting in serious economic losses [3,4,5,6]. ‘Mingrijian’ (M), also referred to as ‘Asumi’, originated in Japan. It is a mandarin hybrid resulting from the crossbreeding of ‘Harumi’ (*Citrus reticulata* × (*Citrus reticulata* × *Citrus sinensis*)) and ‘Sweet spring’ (*Citrus reticulata* cv. Unshiu × *Citrus Hassaku* Hort. ex Tanaka). This hybrid is known for its thin easily peeled skin and high soluble solids content, thus it has gained popularity among consumers and has been recognized as a promising new cultivar in the citrus industry. However, the fruit is susceptible to cracking during its expanding period, which severely constrains the development of this cultivar.

Sudden heavy rainfall following an extended drought can increase the susceptibility of citrus fruits to cracking, a phenomenon that is often observed in the rapid expanding period of fruits. This phenomenon mainly stems from the uncoordinated growth between the pulp and pericarp. After rainfall, the fruit pulp rapidly absorbs water and expands. If the internal expansion pressure exceeds the pericarp strength, fruit cracking will occur.

The mechanical properties of the pericarp play a major role in resisting internal expanding pressure and controlling fruit cracking. As the fruit ripens, cell wall components change and degrade, leading to alterations in the cell wall structure and consequently affecting the mechanical properties of the pericarp [7,8,9]. During this process, the fruit is more susceptible to cracking, highlighting the significant role of cell wall components in the fruit-cracking process. The primary components of the cell wall include polysaccharides, such as pectin, cellulose, and hemicellulose, as well as lignin, proteins, and minerals. Previous research has revealed that during the maturation of jujube fruits, the content of protopectin and cellulose in the peel gradually decreased, while the content of water-soluble pectin (WSP) increased, which could potentially contribute to the increasing rate of fruit cracking [10]. In a comparative study on cracking-susceptible and cracking-resistant cultivars, the susceptible litchi cultivar ‘Nuomici’ had lower protopectin and cellulose contents in the cell wall of the pericarp than the resistant cultivar ‘Huaizhi’ [11]. Similarly, in tomato, the resistant cultivar ‘LA1698’ had higher protopectin and hemicellulose contents in the fruit than the susceptible cultivar ‘LA2683’ [12].

The susceptibility to cracking is closely linked to moisture absorption by the pulp, which becomes the main source of pressure on the pericarp. Osmoregulation serves as a critical regulatory mechanism in plants to maintain cellular osmotic pressure, and it subsequently influences levels of moisture absorption [13]. Soluble sugar (SS) is one of the main osmoregulatory substances in fruit. Previous studies have shown that exogenous nordihydroguaiaretic acid (NDGA) treatment could significantly reduce the contents of SS and soluble solids in grapefruits, thereby decreasing osmotic pressure, water uptake, and peel expansion and inhibiting fruit cracking [14]. According to Richardson et al. [15], sweet cherry fruits containing high SS levels typically experienced elevated osmotic pressure, which affected both moisture absorption and movement, ultimately leading to fruit cracking. Additionally, certain osmoregulatory substances can also enhance the stress resistance of plants. For example, plants protect themselves from the oxidative damage caused by dehydration by proactively accumulating soluble protein (SP) and respond to external stress by increasing reactive oxygen species (ROS), which inflict damage upon the cellular membrane system. Excessive accumulation of harmful substances leads to the occurrence of fruit cracking [16]. The response of the antioxidant enzyme system can reduce membrane damage and enhance stress resistance [17]. Antioxidant enzymes include superoxide dismutase (SOD), catalase (CAT), and peroxidase (POD), among others [18].

**Figure 1:**
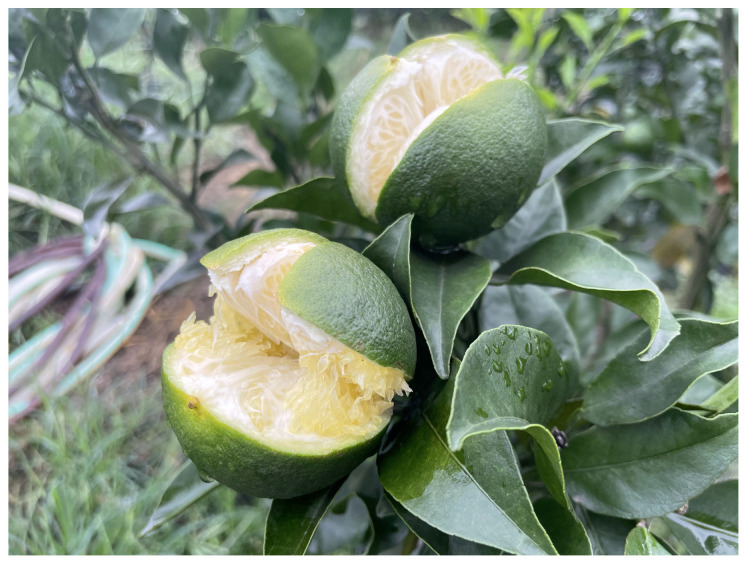
Diagram of fruit cracking of ‘Mingrijian’.

While preliminary research progress has been made on citrus fruit cracking, previous studies mainly focused on certain varieties, fruit morphologies (e.g., peel thickness and anatomical structure), cell wall components, and mineral elements [4,19]; thus, the underlying mechanism remains unclear. In ‘Mingrijian’, fruit cracking mainly happens at the top of the fruit, where the exocarp and mesocarp rapidly crack within a few minutes, leading to juice cell exposure and eventual fruit abscission (Figure 1). Comparative analyses between cracking and noncracking pericarps within a single citrus cultivar are difficult to perform because uncertain factors that arise after the occurrence of cracking, such as emergency response, oxidative stress, and pathogenic invasion, would interfere with the measured results. Local cultivation experience and preliminary laboratory surveys revealed that the citrus cultivar ‘Daya’ (D) hardly ever cracked, while ‘Mingrijian’ (M) cracked at a rate exceeding 60% in regions experiencing severe cracking. Both ‘Daya’ and ‘Mingrijian’ share certain traits, including a relatively thin peel, high soluble solids content in the pulp, and similar maturation periods in suitable planting areas. Therefore, this study utilized the cracking-susceptible cultivar ‘Mingrijian’ and the cracking-resistant cultivar ‘Daya’ as materials to conduct a comparative analysis of biochemical and metabolic changes primarily related to cell wall components, osmoregulatory substances, and antioxidant enzymes. The objective was to explore the contributions of these factors to fruit cracking and reveal the important elements that influence the pericarp mechanical properties and pulp water absorption capacity. This research contributes to elucidating the mechanism of citrus fruit cracking, thereby establishing a foundation for identifying effective prevention and control strategies.

## 2. Materials and Methods

### 2.1. Materials

The samples were collected from Renshou County, Meishan City, Sichuan Province, China. A total of 15 healthy trees from both the ‘Mingrijian’ and ‘Daya’ citrus cultivars with uniform growth were selected, and the planting density was 3 m × 5 m. Each group of five trees formed a designated plot and replicate, thus ensuring uniform cultivation practices across all units. Four representative time points were selected based on preliminary laboratory statistics of fruit cracking rates for ‘Mingrijian’: before fruit cracking (75 days after flowering), initial stage of fruit cracking (115 days after flowering), peak stage of fruit cracking (150 days after flowering), and end stage of fruit cracking (180 days after flowering). Samples were collected separately from ‘Mingrijian’ and ‘Daya’ at each designated time point. From the periphery of the canopy, a total of thirty disease-free fruits of consistent size were carefully selected from each cultivar, covering all four cardinal directions: east, south, west, and north. After returning the fruits to the laboratory, they were cleaned, and the pericarp and pulp were separated, with a portion stored at −80 °C for measurements of osmoregulatory substances and enzyme activities, and the remaining portion underwent a drying process at 80 °C for 72 h for analysis of cell wall components.

### 2.2. Extraction and Determination of Cell Wall Material

Cell wall material (CWM) was extracted according to Huber et al. [20] with modifications. The dried pericarp tissue was crushed and sieved. A sample (1 g) was weighed and placed into a centrifuge tube. Then, 30 mL of 80% ethanol solution was added, and the tube was placed in a boiling water bath for 25 min. After cooling and vacuum filtration, the filter residue was rinsed three times with 30 mL of 80% ethanol solution and then collected. The collected residue was soaked in 30 mL of 90% dimethylsulfoxide solution, left overnight, and then vacuum-extracted again. The residue was rinsed with 30 mL acetone three times, dried to obtain CWM, and then weighed. The amount of CWM obtained per gram of sample was calculated. Three replicates were set up for each sample.

### 2.3. Determination of Cell Wall Polysaccharides

The separation of CWM was conducted according to Siddiqui et al. [21] with modifications. A CWM sample (0.01 g) was accurately weighed, and the protopectin content was determined using the protopectin test kit (Product No. G0703F; Suzhou Grace Biotechnology Co., Ltd., Suzhou, China). Additionally, 0.05 g of CWM was mixed with 5 mL of 50 mmol·L^−1^ sodium acetate buffer (pH 6.5), shaken for 6 h, and centrifuged at 9500 rpm for 10 min to obtain the WSP supernatant. Separately, 5 mL of 50 mmol·L^−1^ sodium acetate buffer (containing 50 mmol·L^−1^ EDTA, pH 6.5) was added to the sediment, shaken for 6 h, and then centrifuged at 9500 rpm for 10 min to obtain the ionic bound pectin (ISP) supernatant; and 5 mL of 50 mmol·L^−1^ Na_2_CO_3_ solution (containing 2 mmol·L^−1^ EDTA) was added to the sediment, shaken for 6 h, and then centrifuged at 9500 rpm for 10 min to obtain the supernatant containing covalently bound pectin (CSP). The contents of pectin (WSP, ISP, CSP) were determined by the carbazole colorimetric method [22].

After adding 5 mL of 4 mmol·L^−1^ NaOH solution (including 100 mmol·L^−1^ NaBH_4_) to the sediment, the mixture was shaken for 6 h and then centrifuged at 9500 rpm for 10 min to obtain the supernatant containing hemicellulose. A 2 mL portion of the supernatant was collected, combined with 3 mL of 2 mol·L^−1^ sulfuric acid, and hydrolyzed at 100 °C in a boiling water bath for 5 h. The hydrolyzed reducing sugars were determined using the anthrone method. The sediment obtained from the previous step was cellulose, which was mixed with 1.5 mL of 80% sulfuric acid, left for 2 h, then mixed with 3 mL of water and hydrolyzed at 100 °C in a boiling water bath for 5 h. The hydrolyzed reducing sugars were determined using the anthrone method [23].

### 2.4. Determination of Lignin

The lignin content was determined by the acetylation method using a lignin test kit (Product No. G0708W; Suzhou Grace Biotechnology Co., Ltd., Suzhou, China).

### 2.5. Determination of Osmoregulatory Substances

The SS content was determined by anthrone colorimetry [24], and the SP content was determined by the Coomassie brilliant blue method [25].

### 2.6. Determination of Antioxidant Enzyme Activity

SOD activity (U/g FW) was determined by the NBT photoreduction method [26], where the amount required to inhibit the photochemical reduction of NBT to 50% of the control was defined as 1 unit of enzyme activity (U). POD activity (U/g FW) was determined by the guaiacol method [27], where a change of 0.01 in A470 within 1 min was defined as 1 unit of enzyme activity (U). CAT activity (U/g FW) was determined by UV spectrophotometry [27], where a decrease of 0.1 in A240 within 1 min was defined as 1 unit of enzyme activity (U).

### 2.7. Statistical Analysis

The experimental data underwent analysis employing an independent samples *t*-test through IBM SPSS Statistics 23.0. Duncan’s multiple-range test was utilized to assess differences between the samples, with statistical significance set at *p* < 0.05. Furthermore, a correlation analysis was conducted using SPSS 23.0 and R 3.5.3. Graphs were generated using Excel 2016, R 3.5.3, and Adobe Illustrator 2020.

## 3. Results

### 3.1. Changes in the Cell Wall Components and Contents in the Pericarp during Fruit Development in ‘Mingrijian’ and ‘Daya’

#### 3.1.1. Changes in Cell Wall Material Content

**Figure 2:**
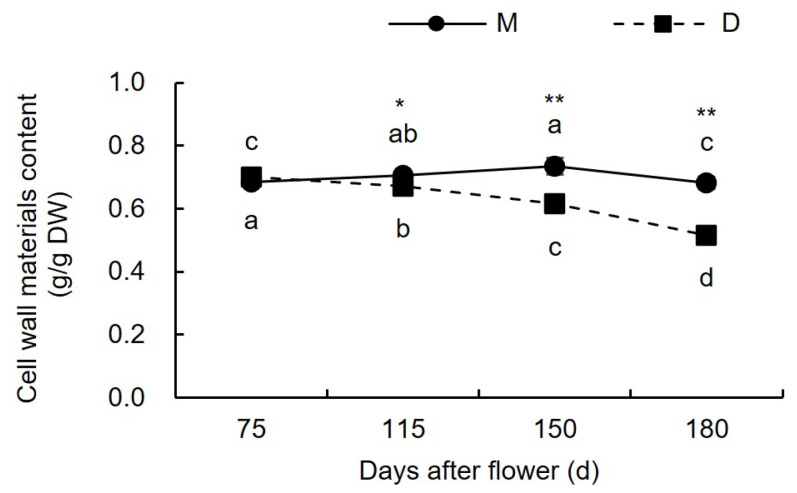
Changes in the cell wall material in the pericarp of ‘Mingrijian’ and ‘Daya’ during fruit development. Different lowercase letters represent significant differences in the different growth and development stages at the 0.05 level (*p* < 0.05). * Significant difference between two varieties (*p* < 0.05); ** extremely significant difference between two varieties (*p* < 0.01).

Figure 2 shows that from 75 to 180 days after flowering, the content of CWM in the pericarp of ‘Mingrijian’ initially increased, reached its maximum at the peak cracking stage (150 days after flowering), and then decreased. The CWM content in the pericarp of ‘Daya’ gradually decreased from 0.70 g/g dry weight (DW) to 0.52 g/g DW. The results of the variance analysis indicated that the CWM content did not significantly differ between the pericarps of ‘Daya’ and ‘Mingrijian’ at 75 days after flowering. However, the CWM content in the pericarp of ‘Daya’ consistently remained lower than that of ‘Mingrijian’ during the period from 115 to 180 days after flowering.

#### 3.1.2. Changes in Pectin Content

**Figure 3:**
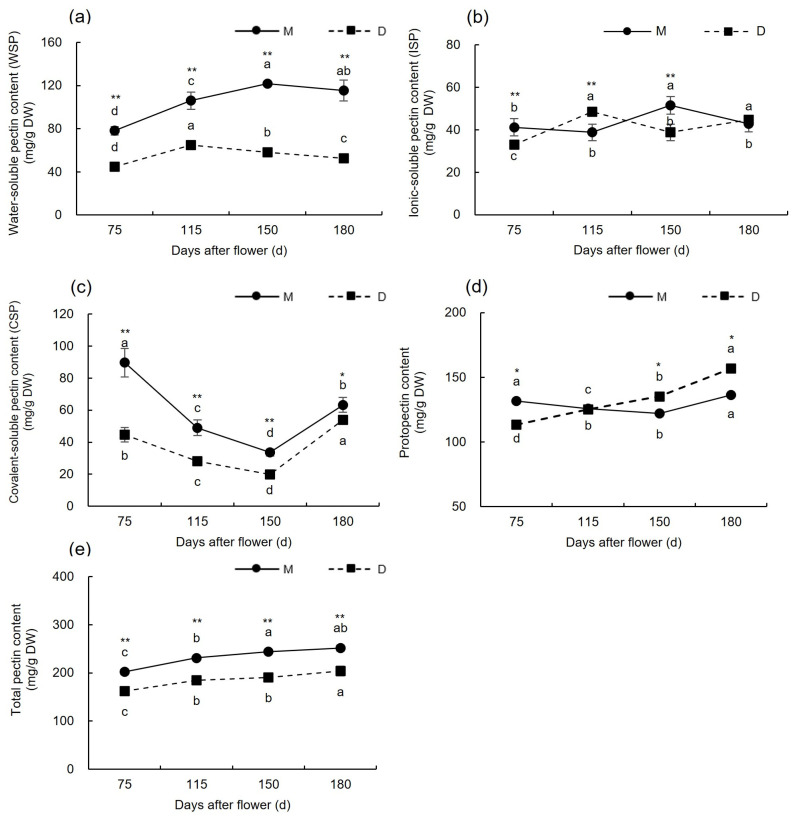
Changes in pectin contents in the pericarp of ‘Mingrijian’ and ‘Daya’ during fruit development. (**a**) Water-soluble content; (**b**) ionic-soluble pectin content; (**c**) covalent-soluble pectin content; (**d**) protopectin content; and (**e**) total pectin content. Different lowercase letters represent significant differences in the different growth and development stages at the 0.05 level (*p* < 0.05). * Significant difference between two varieties (*p* < 0.05); ** extremely significant difference between two varieties (*p* < 0.01).

As shown in Figure 3a, before the occurrence of cracking (75–115 days after flowering), the WSP content in the pericarp of both ‘Mingrijian’ and ‘Daya’ showed an increasing trend at 35.46% and 40.43%, respectively. During the period of fruit cracking from the initial to peak stage (115–150 days after flowering), the WSP content in the pericarp of ‘Mingrijian’ increased from 105. 96 mg/g DW to 121.77 mg/g DW as the fruit cracking rate increased, whereas that of ‘Daya’ decreased from 64.71 mg/g DW to 58.34 mg/g DW. From the peak to end stage of fruit cracking (150–180 days after flowering), the WSP content in the pericarp of both ‘Mingrijian’ and ‘Daya’ showed a decreasing trend, with similar rates of decrease. As shown in Figure 3b, the ISP content in the pericarp of ‘Mingrijian’ and ‘Daya’ exhibited fluctuating trends from 75 to 180 days after flowering, characterized by a ‘decreasing-increasing-decreasing’ pattern for ‘Mingrijian’ and an ‘increasing-decreasing-increasing’ pattern for ‘Daya’. As shown in Figure 3c, the CSP and WSP contents in the pericarp of ‘Mingrijian’ exhibited contrasting patterns of change during the same period. From the initial to peak stage of fruit cracking (115–150 days after flowering), the CSP content in the pericarp of ‘Mingrijian’ decreased by 31.41% as the cracking rate increased. As shown in Figure 3d, from the pre-cracking to peak stage (75–150 days after flowering), the protopectin content in the pericarp of ‘Mingrijian’ continuously declined, reaching a minimum value of 122.03 mg/g DW at 150 days after flowering, while that in ‘Daya’ generally increased by 38.52%. As shown in Figure 3e, from 75 to 180 days after flowering, the total pectin content in the pericarp of both ‘Mingrijian’ and ‘Daya’ showed an increasing trend. Throughout this period, the contents of total pectin, WSP, and CSP in the pericarp of ‘Daya’ were significantly lower than those in ‘Mingrijian’. However, after the occurrence of cracking (115–180 days after flowering), the protopectin content in the pericarp of ‘Daya’ exceeded that of ‘Mingrijian’. The results indicated a mutual transformation between protopectin (predominantly CSP) and WSP in the pericarp of ‘Mingrijian’ in response to fruit cracking.

#### 3.1.3. Changes in Cellulose and Hemicellulose Contents

**Figure 4:**
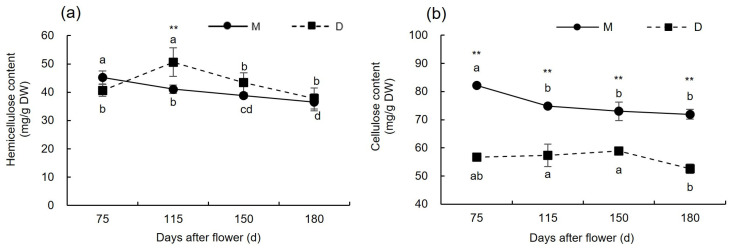
Changes in cellulose and hemicellulose contents in the pericarp of ‘Mingrijian’ and ‘Daya’ during fruit development. (**a**) Hemicellulose content; and (**b**) cellulose content. Different lowercase letters represent significant differences in the different growth and development stages at the 0.05 level (*p* < 0.05). ** Extremely significant difference between two varieties (*p* < 0.01).

As shown in Figure 4a, the hemicellulose content in the pericarp of ‘Mingrijian’ decreased by 9.21% before the occurrence of fruit cracking (75–115 days after flowering), while that in ‘Daya’ significantly increased by 24.53%. This substantial increase in hemicellulose content in the pericarp of ‘Daya’ caused its hemicellulose levels to surpass those of ‘Mingrijian’ from 115 to 180 days after flowering. Notably, at 115 days after flowering, ‘Daya’ exhibited significantly higher hemicellulose content than ‘Mingrijian’. From the initial to end stage of fruit cracking (115–180 days after flowering), both ‘Mingrijian’ and ‘Daya’ showed a decreasing trend in hemicellulose content. As shown in Figure 4b, no significant differences in cellulose content were observed in the pericarp of ‘Mingrijian’ with the occurrence of fruit cracking. 

#### 3.1.4. Changes in Lignin Content

**Figure 5:**
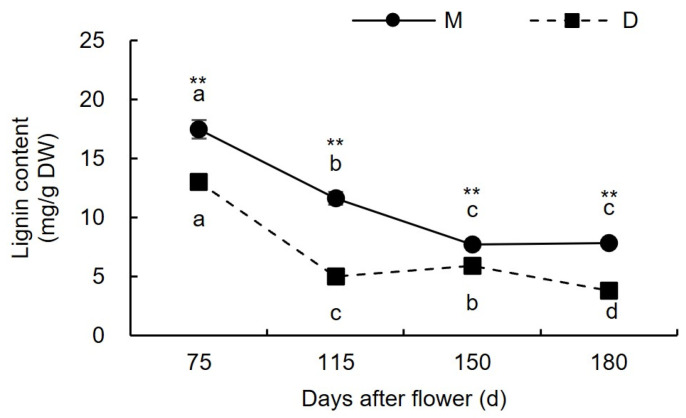
Changes in lignin content in the pericarp of ‘Mingrijian’ and ‘Daya’ during fruit development. Different lowercase letters represent significant differences in the different growth and development stages at the 0.05 level (*p* < 0.05). ** Extremely significant difference between two varieties (*p* < 0.01).

As illustrated in Figure 5, before the occurrence of cracking (75–115 days after flowering), the lignin content in the pericarp of both ‘Mingrijian’ and ‘Daya’ decreased. From the initial to peak stage of fruit cracking (115–150 days after flowering), the lignin content in the pericarp exhibited contrasting trends in ‘Mingrijian’ and ‘Daya’. Specifically, ‘Mingrijian’ displayed a significant reduction from 11.63 mg/g DW to 7.72 mg/g DW, representing a decrease of 33.64%, whereas ‘Daya’ exhibited a notable increase from 5.03 mg/g DW to 5.92 mg/g DW, representing a rise of 17.63%. During the peak to end stage of fruit cracking (150–180 days after flowering), the lignin content in the pericarp showed no significant change in ‘Mingrijian’ but decreased to 3.82 mg/g DW in ‘Daya’. These results indicated that with the occurrence of fruit cracking, lignin gradually degraded in the pericarp of ‘Mingrijian’.

#### 3.1.5. Correlation Analysis between Cell Wall Components and Fruit Cracking Rate

**Figure 6:**
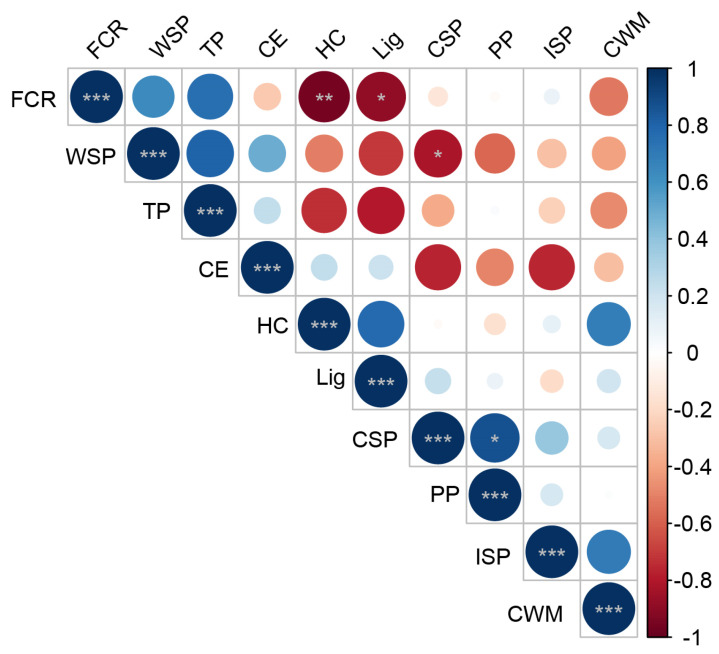
Heat map of the correlations between cell wall components and fruit cracking rate in ‘Mingrijian’. Significance was evaluated using the *t*-test (* *p* < 0.05, ** *p* < 0.01, and *** *p* < 0.001); color depth represents the strength of the correlation; blue indicates the positive correlation, and red indicates the negative correlation. FCR: fruit cracking rate, WSP: water-soluble pectin, TP: total pectin, CE: cellulose, HC: hemicellulose, Lig: lignin, CSP: covalently bound pectin, PP: protopectin, ISP: ionic bound pectin, CWM: cell wall material.

The cell wall components and contents were determined at 75, 95, 115, 135, 150, 165, and 180 days after flowering in the pericarp of ‘Mingrijian’. Subsequently, a correlation analysis was performed between these measurements and the cracking rate data. The results are presented in Figure 6. Significance was evaluated using the *t*-test (* *p* < 0.05). A significant negative correlation was observed between CSP and WSP (R^2^ = −0.819 *), while a significant positive correlation was found between CSP and protopectin (R^2^ = 0.872 *), suggesting a mutual transformation of pectin forms within the pericarp, particularly involving protopectin (mainly CSP) and WSP. The rate of fruit cracking exhibited either a significant or highly significant negative correlation with hemicellulose and lignin (R^2^ ≤ −0.888 *), indicating that the occurrence of fruit cracking in ‘Mingrijian’ was associated with the degradation of hemicellulose and lignin. 

### 3.2. Variations in Osmoregulatory Substances during Fruit Development of ‘Mingrijian’ and ‘Daya’

#### 3.2.1. Variations in Soluble Sugar Content in the Fruit

**Figure 7:**
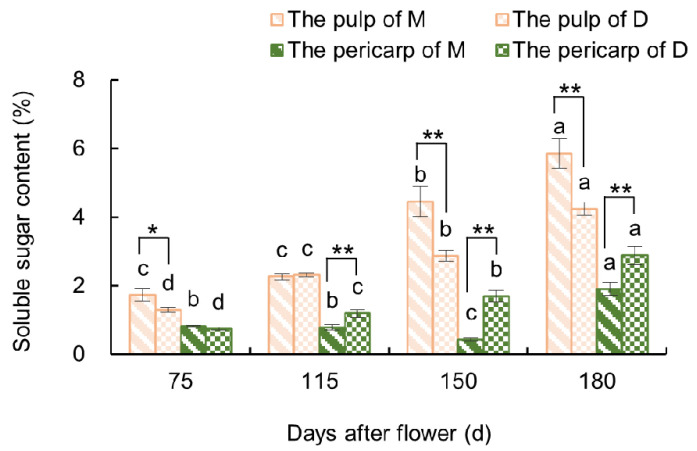
Changes in soluble sugar content in the fruit of ‘Mingrijian’ and ‘Daya’ during fruit development. Bars represent the SE (±), and asterisks indicate a significant difference; * significant difference between two varieties (*p* < 0.05); ** extremely significant difference between two varieties (*p* < 0.01). Different lowercase letters represent significant differences in the different growth and development stages at the 0.05 level (*p* < 0.05).

As shown in Figure 7, before the occurrence of fruit cracking (75–115 days after flowering), the SS content did not significantly vary in the pulp of ‘Mingrijian’. However, from the initial to peak stage of fruit cracking (115–150 days after flowering), there was a significant rise in the SS content in the pulp of ‘Mingrijian’ from 2.26% to 4.46%, which represented a substantial increase of 96.97%. This value was significantly higher than that of ‘Daya’ at 150 days after flowering. From the peak to end stage of fruit cracking (150–180 days after flowering), both ‘Mingrijian’ and ‘Daya’ exhibited a continuous rise in SS content in their pulp. As for the pericarp, the SS content in ‘Daya’ gradually increased from 0.74% to 2.89% from 75 to 180 days after flowering, which was similar to the variation observed in the pulp. However, the SS content in ‘Mingrijian’ remained at a comparatively low level from 75 to 180 days after flowering, ranging from 0.44% to 1.90%. These results indicated a notable rise in the SS content in the pulp of ‘Mingrijian’ as the rate of fruit cracking rapidly increased (115–150 days after flowering).

#### 3.2.2. Variations in Soluble Protein Content in the Fruit

**Figure 8:**
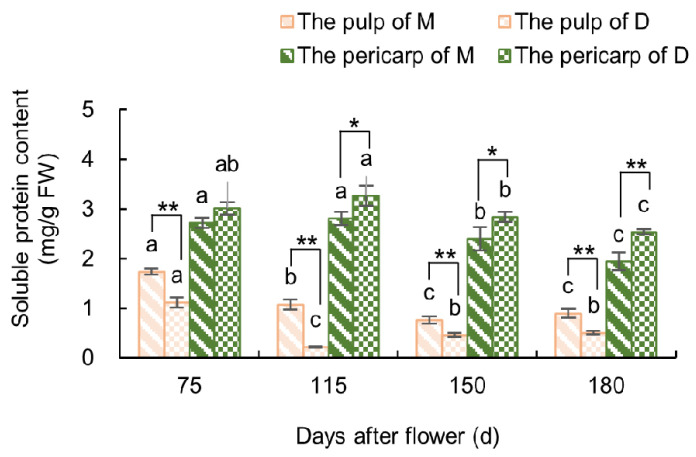
Changes in soluble protein content in the fruit of ‘Mingrijian’ and ‘Daya’ during fruit development. Bars represent the SE (±), and asterisks indicate a significant difference; * significant difference between two varieties (*p* < 0.05); ** extremely significant difference between two varieties (*p* < 0.01). Different lowercase letters represent significant differences in the different growth and development stages at the 0.05 level (*p* < 0.05).

As shown in Figure 8, from the initial to peak stage of fruit cracking (115–150 days after flowering), the SP content in the pulp of ‘Mingrijian’ was significantly decreased by 28.63%, while that in the pulp of ‘Daya’ was significantly increased by 113.07%. Moreover, significant reductions in SP content were observed in the pericarp in both ‘Mingrijian’ and ‘Daya’ from 115 to 180 days after flowering. Following fruit cracking (115–180 days after flowering), the SP content in the pericarp of ‘Mingrijian’ was 16.15–30.85% lower than that of ‘Daya’. The results showed that there was a significant and noticeable decrease in both the pulp and pericarp SP content in ‘Mingrijian’ as the rate of fruit cracking rapidly increased (115–150 days after flowering).

### 3.3. Changes in Antioxidant Enzyme Activities in the Pericarp during Fruit Development in ‘Mingrijian’ and ‘Daya’

#### 3.3.1. Changes in Superoxide Dismutase Activity

**Figure 9:**
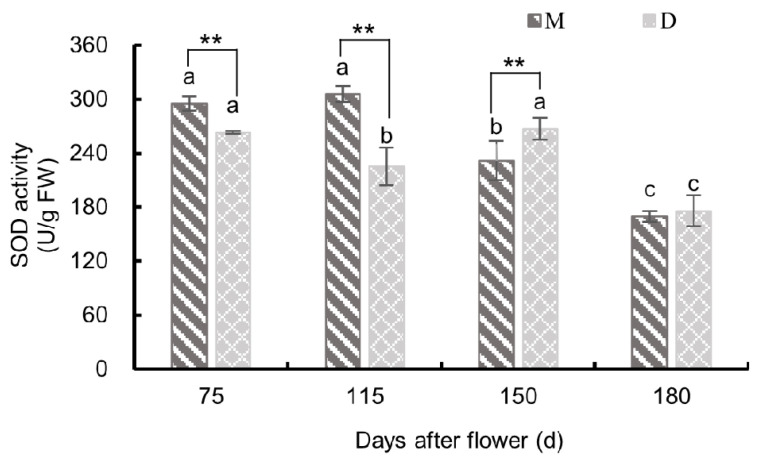
Changes in superoxide dismutase (SOD) activity in the pericarp of ‘Mingrijian’ and ‘Daya’ during fruit development. Bars represent the SE (±), and asterisks indicate a significant difference; ** extremely significant difference between two varieties (*p* < 0.01). Different lowercase letters represent significant differences in the different growth and development stages at the 0.05 level (*p* < 0.05).

As shown in Figure 9, the pericarp of both ‘Mingrijian’ and ‘Daya’ exhibited distinct patterns in SOD activity from 75 to 180 days after flowering. ‘Mingrijian’ displayed an initial increase followed by a subsequent decrease, while ‘Daya’ showed a fluctuating ‘decreasing-increasing-decreasing’ trend. Notably, before the occurrence of fruit cracking (75–115 days after flowering), the SOD activity in the pericarp of ‘Mingrijian’ significantly exceeded that of ‘Daya’. From the initial to peak cracking stage (115–150 days after flowering), the SOD activity in the pericarp of ‘Mingrijian’ decreased significantly from 305.19 U/g FW to 231.71 U/g FW, representing a reduction of 24.08%, whereas that in ‘Daya’ showed a significant increase from 225.3 U/g FW to 267.07 U/g FW, representing an increase of 18.49%. Due to these changes, the SOD activity in the pericarp of ‘Mingrijian’ was notably lower than that in ‘Daya’ at 150 days after flowering. From the peak to end stage of fruit cracking (150–180 days after flowering), a distinct decline in SOD activity was observed in the pericarp of both ‘Mingrijian’ and ‘Daya’, with reductions of 26.77% and 34.16%, respectively. These findings indicated a significant reduction in SOD activity in the pericarp of ‘Mingrijian’ as the rate of fruit cracking rapidly increased (115–150 days after flowering). 

#### 3.3.2. Changes in Peroxidase Activity

**Figure 10:**
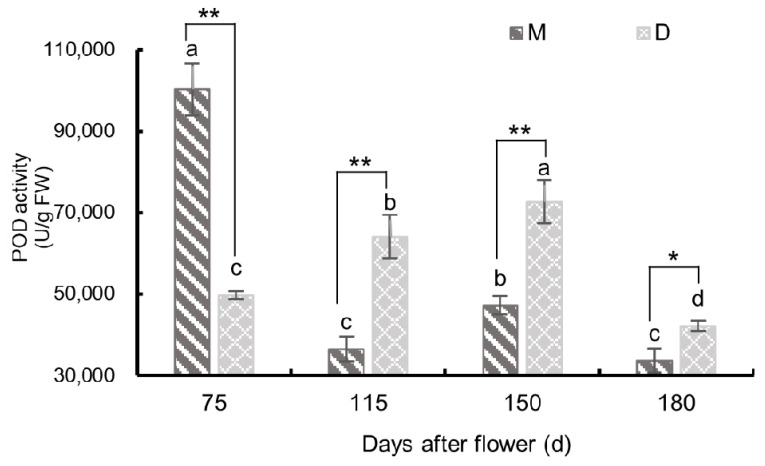
Changes in peroxidase (POD) activity in the pericarp of ‘Mingrijian’ and ‘Daya’ during fruit development. Bars represent the SE (±), and asterisks indicate a significant difference; * significant difference between two varieties (*p* < 0.05); ** extremely significant difference between two varieties (*p* < 0.01). Different lowercase letters represent significant differences in the different growth and development stages at the 0.05 level (*p* < 0.05).

As shown in Figure 10, the POD activity in the pericarp of both ‘Mingrijian’ and ‘Daya’ exhibited distinctive patterns from 75 to 180 days after flowering. ‘Mingrijian’ exhibited a ‘decreasing-increasing-decreasing’ trend, while ‘Daya’ displayed an initial increase followed by a decreasing trend. Notably, before fruit cracking (75 days after flowering), the POD activity in the pericarp of ‘Mingrijian’ was 100,235.28 U/g FW, which was significantly higher (2.02 times) than that of ‘Daya’. From 75 to 115 days after flowering, the POD activity in the pericarp of ‘Mingrijian’ decreased rapidly to 36,449.17 U/g FW, representing a decrease of 63.64%, whereas that in ‘Daya’ increased to 64,107.54 U/g FW, representing an increase of 28.89%. From the initial to peak stage of fruit cracking (115–150 days after flowering), the POD activity in the pericarp of both ‘Mingrijian’ and ‘Daya’ increased significantly, with ‘Daya’ reaching its peak value of 72,671.78 U/g FW at 150 days after flowering. From 115 to 150 days after flowering, the POD activity in the pericarp of ‘Daya’ was significantly higher than that in ‘Mingrijian’. These results indicated a marked decrease in POD activity in the pericarp of ‘Mingrijian’ before the occurrence of fruit cracking (75–115 days after flowering).

#### 3.3.3. Changes in Catalase Activity

**Figure 11:**
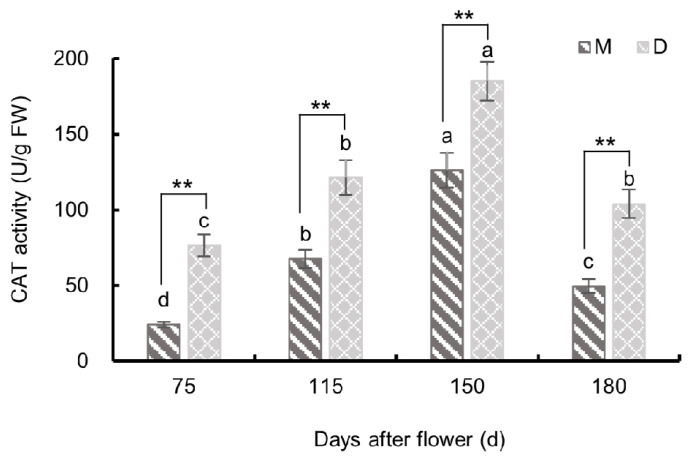
Changes in catalase (CAT) activity in the pericarp of ‘Mingrijian’ and ‘Daya’ during fruit development. Bars represent the SE (±), and asterisks indicate a significant difference; ** extremely significant difference between two varieties (*p* < 0.01). Different lowercase letters represent significant differences in the indexes among different growth and development stages at 0.05 level (*p* < 0.05).

As shown in Figure 11, the CAT activity in the pericarp of both ‘Mingrijian’ and ‘Daya’ showed a distinct pattern, with an initial increase followed by a decrease from 75 to 180 days after flowering. Notably, both cultivars exhibited maximum activity at 150 days after flowering, with values of 126.31 U/g FW for ‘Mingrijian’ and 185.09 U/g FW for ‘Daya’. From 75 to 180 days after flowering, the CAT activity in the pericarp of ‘Mingrijian’ was significantly lower than that of ‘Daya’. These results indicated that with the rapid escalation of the fruit cracking rate, CAT activity in the pericarp increased.

### 3.4. Correlation Analysis of Osmoregulatory Substances, Antioxidant Enzymes, and Fruit Cracking Rates

**Figure 12:**
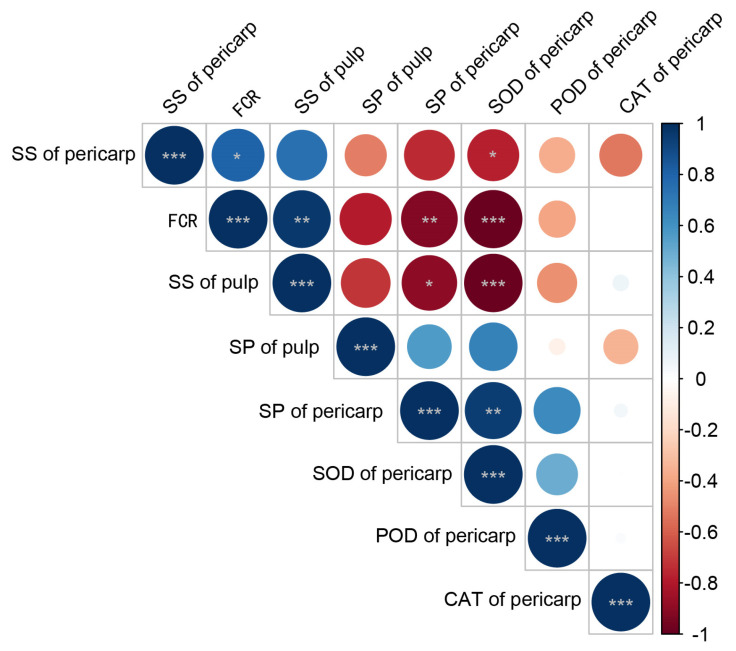
Heat map of the correlation between the fruit cracking rate and osmoregulatory substances and antioxidant enzyme in the fruit of ‘Mingrijian’. Significance was evaluated using the *t*-test (* *p* < 0.05, ** *p* < 0.01, and *** *p* < 0.001); color depth represents the strength of the correlation; blue indicates the positive correlation, and red indicates the negative correlation. SS: soluble sugar, FCR: fruit cracking rate, SP: soluble protein, SOD: superoxide dismutase, POD: peroxidase, CAT: catalase.

The cell wall components and contents were determined at 75, 95, 115, 135, 150, 165, and 180 days after flowering for the pericarp of ‘Mingrijian’. Subsequently, a correlation analysis was performed between these measurements and the cracking rate data. The results are presented in Figure 12. As shown in Figure 12, significance was evaluated using the *t*-test (* *p* < 0.05, and ** *p* < 0.01). A significant positive correlation was observed between the fruit cracking rate and SS content in both the pulp (R^2^ = 0.960 **) and pericarp (R^2^ = 0.791 *), indicating a close relationship between the SS content and fruit cracking. Notably, the rapid increase in pulp SS content had a significant effect on fruit cracking. Conversely, a remarkably significant negative correlation was observed between the fruit cracking rate and the SP content and SOD activity (R^2^ ≤ −0.895 **), emphasizing the substantial impact of SP content and SOD activity on fruit cracking.

## 4. Discussion

### 4.1. Relationship between Cell Wall Material and Fruit Cracking

The components of the cell wall play a crucial role in determining the strength, toughness, and structural integrity of the cell wall and represent key factors in maintaining the mechanical properties of fruit peel [11,12]. Studies on jujube have shown that a reduction in protopectin and cellulose content, coupled with an increase in WSP content, could lead to an increasing rate of fruit cracking [10]. Research on citrus fruits has revealed that cracked fruit exhibited lower levels of protopectin and higher levels of WSP than normal fruit [19]. In studies on grapes, immersing ‘Xiangfei’ grapefruits in a calcium solution effectively suppressed the production of WSP, delayed the degradation of protopectin, stabilized the cell wall structure, and improved the mechanical properties of the peel [28]. In the present study, as the fruit cracking rate rapidly increased (from 115 to 150 days after flowering), the protopectin (mainly CSP) in the pericarp of ‘Mingrijian’ progressively transformed into WSP, while hemicellulose and lignin gradually degraded. These changes probably affected the strength and structural integrity of the cell wall and were correlated closely with the occurrence of fruit cracking. Notably, the rate of fruit cracking exhibited a significant and highly negative correlation with the hemicellulose and lignin content (R^2^ ≤ −0.888 *). Presently, a greater number of research studies have focused on the impact of pectin morphological transformations and cellulose degradation on fruit cracking, whereas few investigations have addressed the connection between lignin and fruit cracking [12,29].

During the growth and development of fruit, the cell wall components of different cultivars exhibited distinct patterns of change. Previous research on tomatoes has shown that the crack-resistant tomato genotype ‘LA1698’ exhibited higher levels of protopectin (SSP and CSP) and cellulose than the cracking-susceptible genotype ‘LA2683’ [12]. In this study, from the initial to peak stage of fruit cracking (from 115 to 150 days after flowering), the cracking-resistant cultivar ‘Daya’ exhibited higher levels of protopectin and hemicellulose in its pericarp than ‘Mingrijian’. Furthermore, a gradual accumulation of lignin was observed in ‘Daya’, which reinforced its cell wall and enhanced the mechanical strength of the pericarp. This adaptation enabled the cultivar to withstand the internal expansion stress generated during the fruit’s growth process. From 75 to 180 days after flowering, the cracking-resistant cultivar ‘Daya’ exhibited markedly lower levels of total pectin, WSP, CSP, cellulose and lignin in its pericarp than the cracking-susceptible cultivar ‘Mingrijian’. However, these results were inconsistent with previous studies on citrus [19], tomato [12], and lychee [11], potentially because of the comparatively lower levels of CWM in the pericarp of ‘Daya’.

### 4.2. Relationship between Osmoregulatory Substances and Fruit Cracking

SS is one of the major osmoregulatory substances in fruits. This study revealed a rapid increase in SS content in the pulp of ‘Mingrijian’ during the critical period of fruit cracking and a highly significant positive correlation between the SS content and fruit cracking rate. The phenomenon is likely attributed to the climatic conditions of the growing area, which is marked by prolonged drought during the rapid expansion phase of fruit. In such circumstances, cells proactively accumulate osmoregulatory substances (solutes) to enhance the plant’s water-holding capacity. The rapid accumulation of SS in ‘Mingrijian’ fruits would lead to an increased osmotic pressure. Consequently, if a sudden rainfall event occurred, the fruit would rapidly absorb a substantial amount of water, ultimately causing fruit cracking. During 115 to 180 days after flowering, it is under the climate condition characterized by alternating high-temperature drought and heavy rainfall. This result is consistent with previous research on fruit, such as sweet cherry [15], wax apple [30], grape [14,31], and tomato [12], which identified elevated SS contents and reduced osmotic potential as contributing factors to fruit cracking. In conclusion, the rapid increase in SS content in the pulp appears to be a crucial factor underlying fruit cracking. 

The majority of SPs in plants are enzymes involved in various metabolic processes and serve as crucial indicators of overall plant metabolism [32]. In this study, the SP content in the pericarp of the cracking-susceptible cultivar ‘Mingrijian’ was found to be lower than that of the cracking-resistant cultivar ‘Daya’. Furthermore, during the critical period of fruit cracking (115–150 days after flowering), the SP content in the fruit of ‘Mingrijian’ exhibited a decreasing trend. The decrease in SP content could be attributed to severe damage to the photosynthetic apparatus and cell membranes, which hindered SP synthesis. The other reason could be that the plants accelerated the decomposition of energy substances to supply energy to the organism [33]. 

### 4.3. Relationship between Antioxidant Enzymes and Fruit Cracking

SOD scavenges ROS generated during the ageing of cells, tissues, or organs, thereby protecting cell membranes and maintaining cellular metabolic balance [10]. In this study, the SOD activity in the pericarp of the cracking-susceptible cultivar ‘Mingrijian’ decreased as the fruit cracking rate increased (115–150 days after flowering), while that in the cracking-resistant cultivar ‘Daya’ increased. This result is inconsistent with previous research [12,34]. The decrease in SOD activity in the pericarp of ‘Mingrijian’ could potentially be related to ROS-induced enzyme inactivation or reduced enzyme synthesis, which leads to an increase in membrane lipid peroxidation in the pericarp and ultimately triggers fruit cracking. In this study, at the peak stage of fruit cracking (150 days after flowering), SOD activity was lower in the pericarp of the cracking-susceptible cultivar ‘Mingrijian’ than in the cracking-resistant cultivar ‘Daya’. This suggests that cracking-resistant cultivars exhibited a greater ability to scavenge oxygen radicals than cracking-susceptible cultivars. Furthermore, cellular senescence adapts more slowly to changes in external factors, such as moisture and light [12]. Previously, the SOD activity in the peel of a cracking-resistant variety of tomatoes was found to be higher than that of the cracking-susceptible variety [35]. Therefore, SOD plays a role in preventing the occurrence of fruit cracking.

POD is an oxidoreductase prevalent in fruit trees and plays a dual role in scavenging ROS and cross-linking the phenolic groups, which affects peel extensibility [35,36]. In this study, the POD activity in the pericarp of ‘Mingrijian’ decreased sharply before the occurrence of fruit cracking (75–115 days after flowering). Additionally, the POD activity in the pericarp of the cracking-susceptible cultivar ‘Mingrijian’ was significantly lower than that of the cracking-resistant cultivar ‘Daya’ from the initial to peak stage of fruit cracking (115–150 days after flowering). These results are consistent with those of a previous study on *Akebia trifoliata* [34]. Higher POD activity in the pericarp could contribute to ROS scavenging and improve the resistance to cracking. However, this result is inconsistent with that of other studies on several other fruits, which indicated that POD activity was significantly higher in cracking-susceptible cultivars than in cracking-resistant cultivars and higher in cracked fruits than in normal fruits [12,35]. This may be attributed to the fact that in the previously studied fruits, POD primarily engages in cross-linking with cell wall phenolic components, resulting in increased POD activity and subsequent cell wall stiffening [36,37], thereby diminishing the mechanical properties of the fruit peel.

CAT can reduce oxidative damage to tissues inflicted by H_2_O_2_ to some extent [38]. In this study, the CAT activity in the pericarp of both cracking-susceptible ‘Mingrijian’ and cracking-resistant ‘Daya’ showed an increasing and then decreasing trend from 75 to 180 days after flowering, with the highest activity at the peak stage (150 days after flowering). This suggests that during the critical period of fruit cracking, the intracellular activity of CAT increases to ease oxidative damage by ROS [18,39]. Moreover, the CAT activity was significantly higher in the cracking-susceptible tomato cultivar ‘LA1698’ than in the same tissues of the cracking-resistant cultivar ‘LA2683’ and was higher at the site where cracking occurred than in the fruit that did not crack [35]. The increase in CAT activity could be a defensive response against oxidative stress. However, further research is needed to clarify the relationship between fruit cracking and CAT.

## 5. Conclusions

Pectin morphological transformations and hemicellulose and lignin degradation in the cell wall of ‘Mingrijian’ fruit pericarp are essential factors influencing fruit cracking. With the occurrence of fruit cracking in ‘Mingrijian’, covalently bound pectin transformed into WSP, leading to a notable decrease in protopectin content, which was concomitant with the degradation of both hemicellulose and lignin. From the initial to end stage of fruit cracking (115–180 days after flowering), the cracking-resistant cultivar ‘Daya’ exhibited higher contents of protopectin and hemicellulose in the pericarp than the cracking-susceptible cultivar ‘Mingrijian’. Furthermore, at 115–150 days after flowering, a marked increase in lignin content was observed in the pericarp of ‘Daya’. Such compositional variations in the cell wall contribute to enhancing the mechanical properties of the pericarp.

The SS content in the pulp, SP content in the pericarp, and SOD and POD activities in the pericarp play a crucial role in fruit cracking susceptibility in ‘Mingrijian’. During the rapid expansion phase of the ‘Mingrijian’ fruit, there was a rapid accumulation of SS in the pulp. This alternation in fruit solutes could facilitate significant water uptake by the pulp, especially in response to sudden rainfall. Consequently, this could lead to peel rupture and fruit cracking. From 115 to 180 days after flowering, a significant decrease in SP content and SOD activity and consistently lower levels of POD activity were observed in the pericarp of ‘Mingrijian’. These changes resulted in a weakened metabolic level, lower cellular antioxidant capacity, and reduced resistance to the external environment, thereby increasing the susceptibility to fruit cracking.

## Data Availability

Data are contained within the article.
